# Supplementary material for: Comparative Risk of Acute Kidney Injury Following Concurrent Administration of Vancomycin with Piperacillin/Tazobactam or Meropenem: A Systematic Review and Meta-Analysis of Observational Studies
Source: Antibiotics (Basel). 2022 Apr 14;11(4):526. doi: 10.3390/antibiotics11040526 (PMC9031739; doi:10.3390/antibiotics11040526)
Supplement: Supplementary file 1 [file antibiotics-11-00526-s001.zip › antibiotics-1645230-supplementary.pdf]

Table S1. Summary of inclusion criteria

**Study design**	Observational studies
**Population**	Adult patients (age ≥ 18 years)<br>Hospitalized for at least 48 hours
**Intervention**	Studies were included if they evaluated the use of the following treatments: vancomycin/piperacillin-tazobactam (V+PT) versus vancomycin/meropenem (V+M)
**Primary Outcomes**	Acute kidney injury (AKI)
**Secondary Outcomes**	Length of stay (LOS) in hospital<br>Renal replacement therapy (RRT)<br>Mortality
**Language of publication**	Only English language publications

Table S2: Newcastle–Ottawa scale for assessing the quality of included studies

Study	Selection				Comparability	Outcome			Score
	Representativeness of the exposed cohort	Selection of the non-exposed cohort	Ascertainment of exposure	Demonstration that outcome of interest was not present at start of study	Comparability of cohorts on the basis of the design or analysis	Assessment of outcome	Was follow-up long enough for outcomes to occur	Adequacy of follow up of cohorts	
Alyami, 2017	✓	✓	✓	✓	✓	✓	✓	✓	8
Rutter, 2019	✓	✓	✓	✓	✓✓	✓	✓	✓	9
Blevins, 2019	-	✓	✓	✓	✓✓	✓	✓	✓	8
Robertson, 2018	✓	✓	✓	✓	✓✓	✓	✓	✓	9
Balcı, 2018	✓	✓	✓	✓	✓✓	✓	✓	✓	9
Mullins, 2018	✓	✓	✓	✓	✓✓	✓	✓	✓	9
Kang, 2019	-	✓	✓	✓	✓✓	✓	✓	✓	8
Cannon, 2018	✓	✓	✓	✓	✓✓	✓	✓	✓	9
Schreier, 2019	-	✓	✓	✓	✓✓	✓	✓	✓	8
Ide, 2019	-	✓	-	✓	✓✓	✓	✓	✓	7
Rungkitwattanakul et al,2021	✓	✓	✓	✓	✓	✓	✓	✓	8
Tookhi et al,2021	✓	✓	✓	✓	✓✓	✓	✓	✓	9

Study or Subgroup	log[Odds Ratio]	SE	vancomycin+piperacillin tazobactam Total	vancomycin +meropenem Total	Weight	Odds Ratio IV, Random, 95% CI
Balcı 2018	1.108563	0.331093	63	69	18.5%	3.03 [1.58, 5.80]
Kang 2019	1.23897	0.463361	74	83	12.6%	3.45 [1.39, 8.56]
Robertson 2018	1.924249	0.771345	85	84	5.9%	6.85 [1.51, 31.06]
Rungkitwattanakul, 2021	1.9502	0.6491	74	67	7.8%	7.03 [1.97, 25.09]
Rutter 2019	0.928219	0.168272	9898	338	28.7%	2.53 [1.82, 3.52]
Schreier 2019	0.405465	0.201731	1540	386	26.5%	1.50 [1.01, 2.23]
**Total (95% CI)**			**11734**	**1027**	**100.0%**	**2.72 [1.82, 4.07]**

Heterogeneity: Tau² = 0.12; Chi² = 11.20, df = 5 (P = 0.05); I² = 55%
Test for overall effect: Z = 4.88 (P < 0.00001)

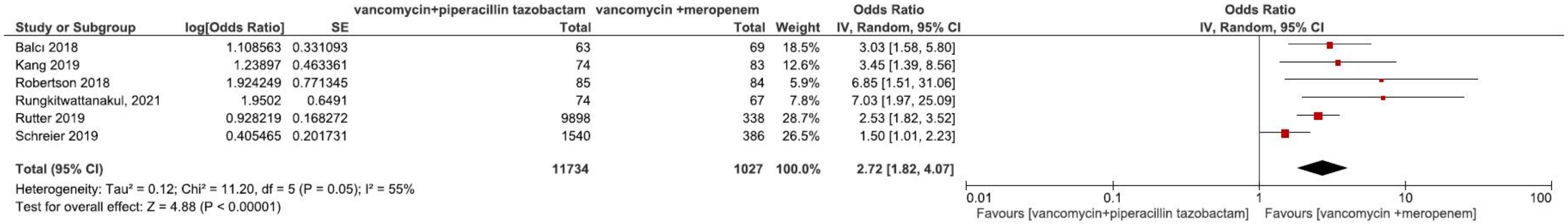

Figure S1. Acute kidney injury (AKI); pooled analysis of the adjusted odds ratio (aOR).

Study or Subgroup	log[Odds Ratio]	SE	vancomycin+piperacillin tazobactam Total	vancomycin +meropenem Total	Weight	Odds Ratio IV, Random, 95% CI
**1.9.1 Non-ICU**						
Balcı 2018	1.108563	0.331093	63	69	17.2%	3.03 [1.58, 5.80]
Ide 2019	1.728109	0.661733	27	49	4.3%	5.63 [1.54, 20.60]
Mullins 2018	0.883768	0.46739	94	47	8.6%	2.42 [0.97, 6.05]
Robertson 2018	1.924249	0.771345	85	84	3.2%	6.85 [1.51, 31.06]
Rutter 2019	0.928219	0.168272	9898	338	66.7%	2.53 [1.82, 3.52]
**Subtotal (95% CI)**			**10167**	**587**	**100.0%**	**2.78 [2.12, 3.64]**
Heterogeneity: Tau² = 0.00; Chi² = 2.97, df = 4 (P = 0.56); I² = 0%						
Test for overall effect: Z = 7.44 (P < 0.00001)						
**1.9.2 ICU**						
Blevins 2019	0.751416	0.15981	366	392	48.3%	2.12 [1.55, 2.90]
Kang 2019	1.23897	0.463361	74	83	12.5%	3.45 [1.39, 8.56]
Schreier 2019	0.405465	0.201731	1540	386	39.2%	1.50 [1.01, 2.23]
**Subtotal (95% CI)**			**1980**	**861**	**100.0%**	**1.97 [1.39, 2.79]**
Heterogeneity: Tau² = 0.04; Chi² = 3.48, df = 2 (P = 0.18); I² = 43%						
Test for overall effect: Z = 3.80 (P = 0.0001)						
**1.9.3 ICU and non-ICU**						
Alyami 2017	0.350657	0.632866	108	75	23.1%	1.42 [0.41, 4.91]
Cannon 2018	1.029619	0.398742	292	74	27.6%	2.80 [1.28, 6.12]
Rungkitwattanakul, 2021	1.9502	0.6491	74	67	22.8%	7.03 [1.97, 25.09]
Tookhi 2021	-0.82098	0.457081	77	81	26.5%	0.44 [0.18, 1.08]
**Subtotal (95% CI)**			**551**	**297**	**100.0%**	**1.81 [0.57, 5.68]**
Heterogeneity: Tau² = 1.08; Chi² = 15.16, df = 3 (P = 0.002); I² = 80%						
Test for overall effect: Z = 1.01 (P = 0.31)						

Test for subgroup differences: Chi² = 2.61, df = 2 (P = 0.27), I² = 23.4%

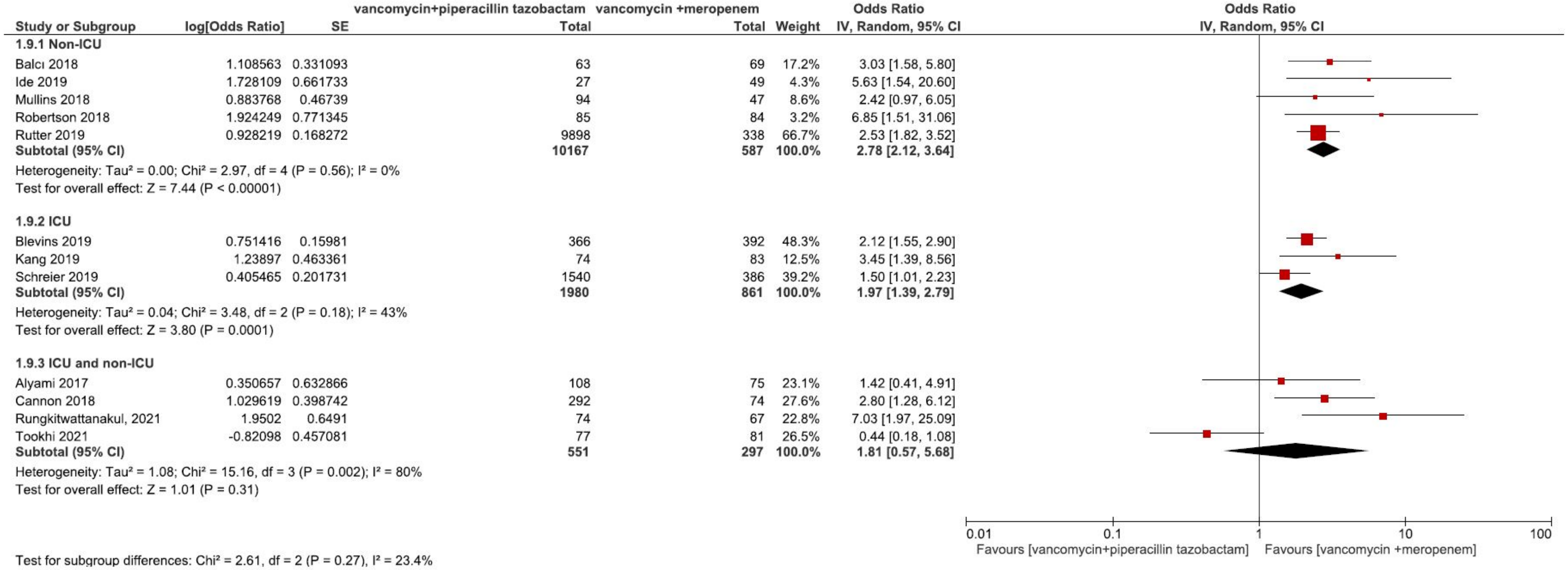

Figure S2. Acute kidney injury (AKI) based on clinical setting.

Study or Subgroup	log[Odds Ratio]	SE	vancomycin+piperacillin tazobactam Total	vancomycin +meropenem Total	Weight	Odds Ratio IV, Random, 95% CI
**1.7.1 ICU**						
Kang 2019	1.23897	0.463361	74	83	37.5%	3.45 [1.39, 8.56]
Schreier 2019	0.405465	0.201731	1540	386	62.5%	1.50 [1.01, 2.23]
**Subtotal (95% CI)**			**1614**	**469**	**100.0%**	**2.05 [0.93, 4.52]**
Heterogeneity: Tau² = 0.22; Chi² = 2.72, df = 1 (P = 0.10); I² = 63%						
Test for overall effect: Z = 1.78 (P = 0.08)						
**1.7.2 NON-ICU**						
Balcı 2018	1.108563	0.331093	63	69	19.8%	3.03 [1.58, 5.80]
Robertson 2018	1.924249	0.771345	85	84	3.6%	6.85 [1.51, 31.06]
Rutter 2019	0.928219	0.168272	9898	338	76.6%	2.53 [1.82, 3.52]
**Subtotal (95% CI)**			**10046**	**491**	**100.0%**	**2.72 [2.04, 3.63]**
Heterogeneity: Tau² = 0.00; Chi² = 1.73, df = 2 (P = 0.42); I² = 0%						
Test for overall effect: Z = 6.79 (P < 0.00001)						
**1.7.3 ICU and non-ICU**						
Rungkitwattanakul, 2021	1.9502	0.6491	74	67	100.0%	7.03 [1.97, 25.09]
**Subtotal (95% CI)**			**74**	**67**	**100.0%**	**7.03 [1.97, 25.09]**
Heterogeneity: Not applicable						
Test for overall effect: Z = 3.00 (P = 0.003)						
Test for subgroup differences: Chi² = 2.63, df = 2 (P = 0.27), I² = 23.9%						

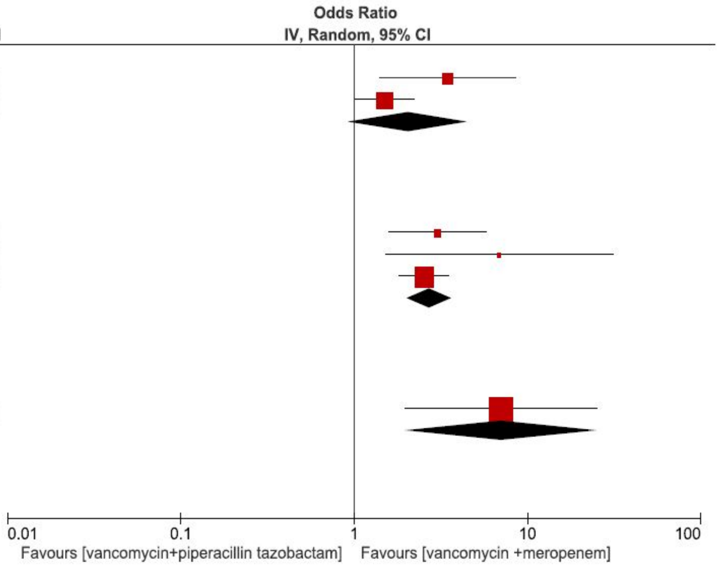

Figure S3. Acute kidney injury (AKI) based on clinical setting for studies reported adjusted analysis for potential confounders.

Study or Subgroup	vancomycin+piperacillin tazobactam Mean	SD	Total	vancomycin +meropenem Mean	SD	Total	Weight	Mean Difference IV, Random, 95% CI
**1.11.1 ICU**								
Robertson 2018	6.15	3.79	85	7.33	4.65	84	47.0%	-1.18 [-2.46, 0.10]
Rutter 2019	11	9.64	9898	10	6.7	338	53.0%	1.00 [0.26, 1.74]
**Subtotal (95% CI)**			**9983**			**422**	**100.0%**	**-0.02 [-2.16, 2.11]**
Heterogeneity: Tau² = 2.09; Chi² = 8.36, df = 1 (P = 0.004); I² = 88%								
Test for overall effect: Z = 0.02 (P = 0.98)								
**1.11.2 NON-ICU**								
Blevins 2019	10.667	8.1868	366	11.25	8.74	392	66.8%	-0.58 [-1.79, 0.62]
Kang 2019	20.5	34.5	74	32	45	83	33.2%	-11.50 [-23.97, 0.97]
**Subtotal (95% CI)**			**440**			**475**	**100.0%**	**-4.20 [-14.28, 5.87]**
Heterogeneity: Tau² = 39.16; Chi² = 2.92, df = 1 (P = 0.09); I² = 66%								
Test for overall effect: Z = 0.82 (P = 0.41)								
**1.11.3 ICU and non-ICU**								
Tookhi 2021	23.5	24.926	77	27.5	26.04	81	100.0%	-4.00 [-11.95, 3.95]
**Subtotal (95% CI)**			**77**			**81**	**100.0%**	**-4.00 [-11.95, 3.95]**
Heterogeneity: Not applicable								
Test for overall effect: Z = 0.99 (P = 0.32)								

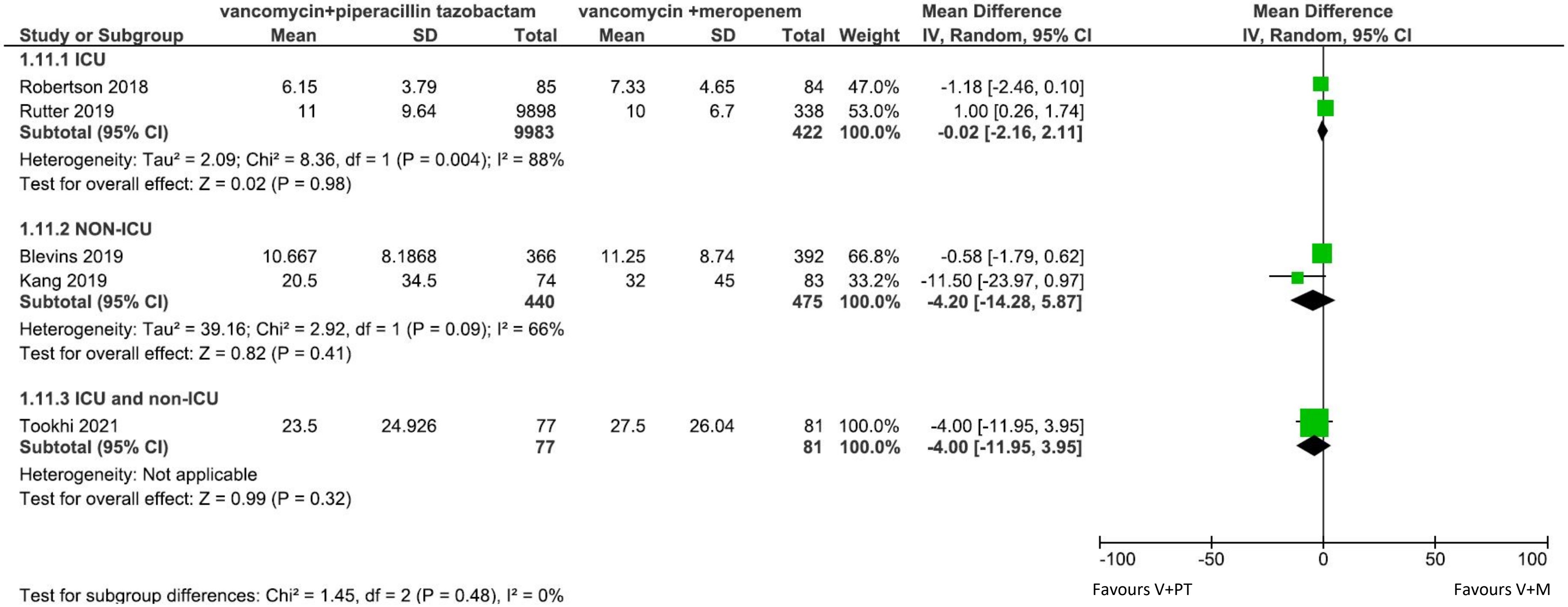

Test for subgroup differences: Chi² = 1.45, df = 2 (P = 0.48), I² = 0%

Figure S4. Length of stay (LOS) in hospital based on clinical setting.

Study or Subgroup	vancomycin+piperacillin tazobactam Events	vancomycin+piperacillin tazobactam Total	vancomycin +meropenem Events	vancomycin +meropenem Total	Weight	Odds Ratio IV, Random, 95% CI
**1.12.1 ICU**						
Blevins 2019	4	366	5	392	71.7%	0.86 [0.23, 3.21]
Schreier 2019	7	604	1	117	28.3%	1.36 [0.17, 11.16]
**Subtotal (95% CI)**		**970**		**509**	**100.0%**	**0.98 [0.32, 2.99]**
Total events	11		6			
Heterogeneity: Tau² = 0.00; Chi² = 0.13, df = 1 (P = 0.71); I² = 0%						
Test for overall effect: Z = 0.04 (P = 0.97)						
**1.12.2 Non-ICU**						
Mullins 2018	3	94	0	47	100.0%	3.63 [0.18, 71.82]
**Subtotal (95% CI)**		**94**		**47**	**100.0%**	**3.63 [0.18, 71.82]**
Total events	3		0			
Heterogeneity: Not applicable						
Test for overall effect: Z = 0.85 (P = 0.40)						

Test for subgroup differences: Chi² = 0.65, df = 1 (P = 0.42), I² = 0%

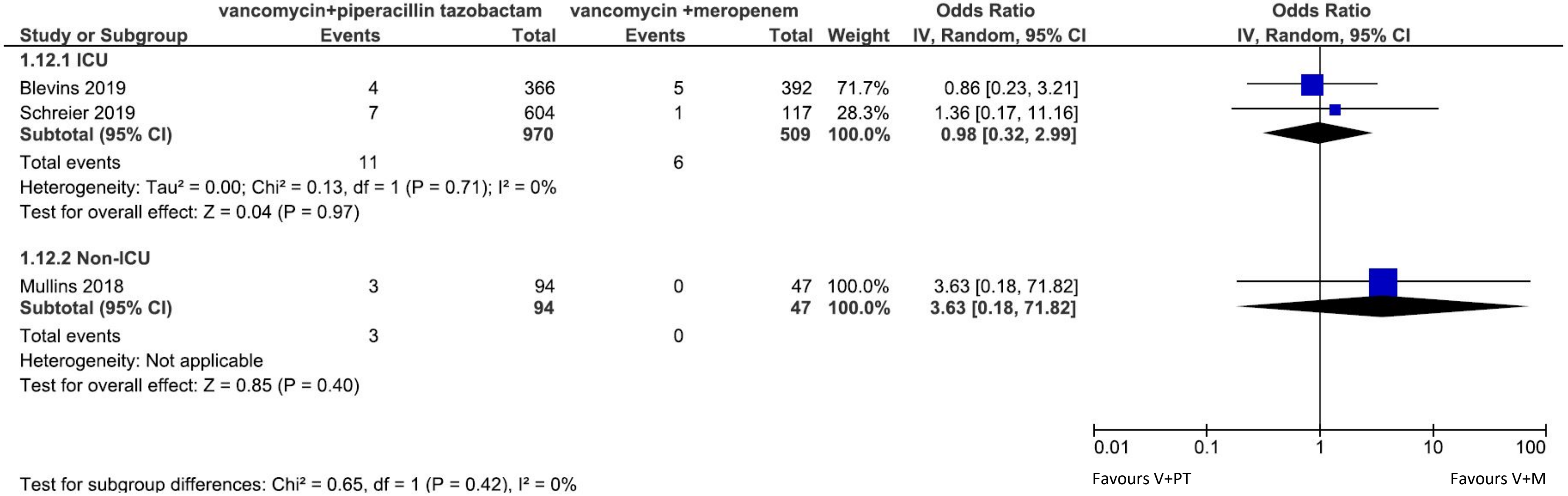

Figure S5. Renal replacement therapy (RRT) based on clinical setting.

	vancomycin+piperacillin tazobactam		vancomycin +meropenem			Odds Ratio
Study or Subgroup	Events	Total	Events	Total	Weight	IV, Random, 95% CI
**1.4.1 Non-ICU**						
Robertson 2018	1	85	0	84	1.2%	3.00 [0.12, 74.69]
Rutter 2019	1144	9898	35	338	98.8%	1.13 [0.79, 1.61]
**Subtotal (95% CI)**		**9983**		**422**	**100.0%**	**1.14 [0.80, 1.63]**
Total events	1145		35			
Heterogeneity: Tau² = 0.00; Chi² = 0.35, df = 1 (P = 0.55); I² = 0%						
Test for overall effect: Z = 0.75 (P = 0.45)						
**1.4.2 ICU**						
Blevins 2019	42	366	67	392	100.0%	0.63 [0.42, 0.95]
**Subtotal (95% CI)**		**366**		**392**	**100.0%**	**0.63 [0.42, 0.95]**
Total events	42		67			
Heterogeneity: Not applicable						
Test for overall effect: Z = 2.19 (P = 0.03)						
**1.4.3 ICU and non-ICU**						
Tookhi 2021	18	77	32	81	100.0%	0.47 [0.23, 0.93]
**Subtotal (95% CI)**		**77**		**81**	**100.0%**	**0.47 [0.23, 0.93]**
Total events	18		32			
Heterogeneity: Not applicable						
Test for overall effect: Z = 2.16 (P = 0.03)						

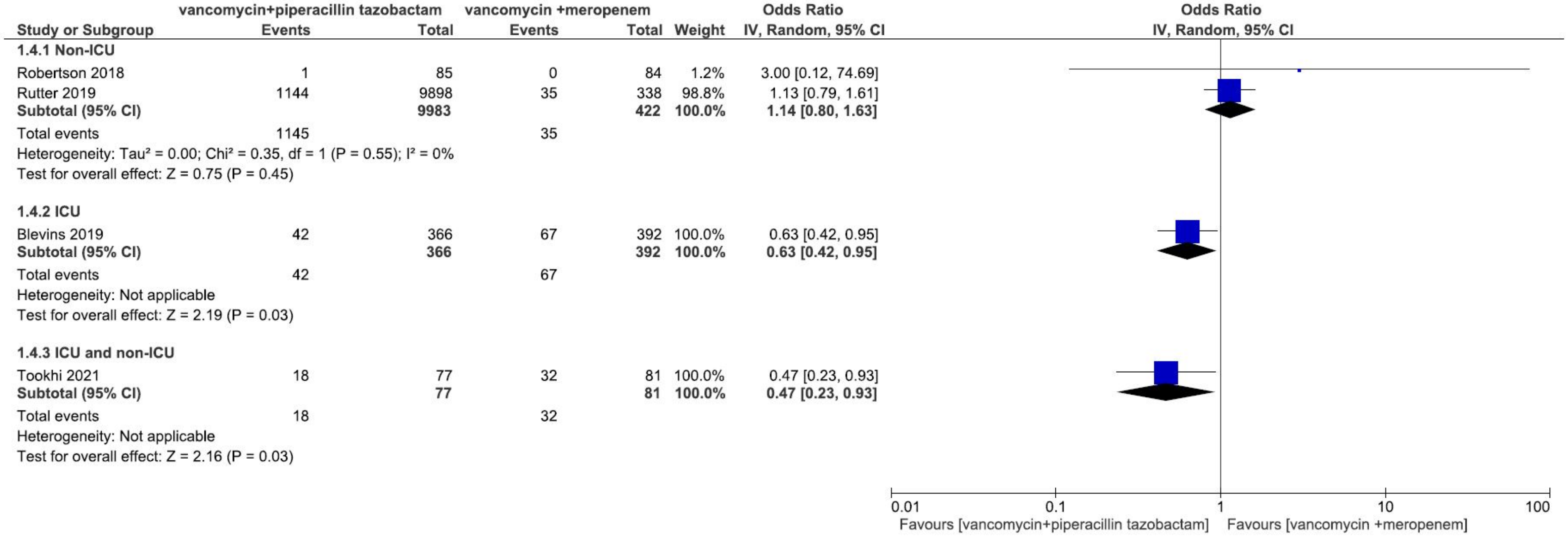

Figure S6. Mortality based on clinical setting.
